# Association of aberrant DNA methylation in Apc^min/+^ mice with the epithelial-mesenchymal transition and Wnt/β-catenin pathways: genome-wide analysis using MeDIP-seq

**DOI:** 10.1186/s13578-015-0013-2

**Published:** 2015-05-27

**Authors:** Yue Guo, Jong Hun Lee, Limin Shu, Ying Huang, Wenji Li, Chengyue Zhang, Anne Yuqing Yang, Sarandeep SS Boyanapalli, Ansu Perekatt, Ronald P Hart, Michael Verzi, Ah-Ng Tony Kong

**Affiliations:** Graduate Program in Pharmaceutical Science, Ernest Mario School of Pharmacy, Rutgers, The State University of New Jersey, Piscataway, NJ 08854 USA; Department of Pharmaceutics, Ernest Mario School of Pharmacy, Rutgers, The State University of New Jersey, Room 228, 160 Frelinghuysen Road, Piscataway, NJ 08854 USA; Department of Genetics, Rutgers, The State University of New Jersey, Piscataway, NJ 08854 USA; Department of Cell Biology and Neuroscience, Rutgers, The State University of New Jersey, Piscataway, NJ 08854 USA; Department of Food Science and Biotechnology, College of Life Science, CHA University, Gyeonggi-do, 463-400 South Korea

**Keywords:** DNA methylation, Epigenetic, MeDIP-seq, Wnt/β-catenin pathway, Epithelial-mesenchymal transition pathway

## Abstract

**Background:**

Aberrant DNA methylation at the 5-carbon on cytosine residues (5mC) in CpG dinucleotides is probably the most extensively characterized epigenetic modification in colon cancer. It has been suggested that the loss of adenomatous polyposis coli (APC) function initiates tumorigenesis and that additional genetic and epigenetic events are involved in colon cancer progression. We aimed to study the genome-wide DNA methylation profiles of intestinal tumorigenesis in Apc^min/+^ mice.

**Results:**

Methylated DNA immunoprecipitation (MeDIP) followed by next-generation sequencing was used to determine the global profile of DNA methylation changes in Apc^min/+^ mice. DNA was extracted from adenomatous polyps from Apc^min/+^ mice and from normal intestinal tissue from age-matched Apc^+/+^ littermates, and the MeDIP-seq assay was performed. Ingenuity Pathway Analysis (IPA) software was used to analyze the data for gene interactions. A total of 17,265 differentially methylated regions (DMRs) displayed a ≥ 2-fold change (log_2_) in methylation in Apc^min/+^ mice; among these DMRs, 9,078 (52.6 %) and 8,187 (47.4 %) exhibited increased and decreased methylation, respectively. Genes with altered methylation patterns were mainly mapped to networks and biological functions associated with cancer and gastrointestinal diseases. Among these networks, several canonical pathways, such as the epithelial-mesenchymal transition (EMT) and Wnt/β-catenin pathways, were significantly associated with genome-wide methylation changes in polyps from Apc^min/+^ mice. The identification of certain differentially methylated molecules in the EMT and Wnt/β-catenin pathways, such as APC2 (adenomatosis polyposis coli 2), SFRP2 (secreted frizzled-related protein 2), and DKK3 (dickkopf-related protein 3), was consistent with previous publications.

**Conclusions:**

Our findings indicated that Apc^min/+^ mice exhibited extensive aberrant DNA methylation that affected certain signaling pathways, such as the EMT and Wnt/β-catenin pathways. The genome-wide DNA methylation profile of Apc^min/+^ mice is informative for future studies investigating epigenetic gene regulation in colon tumorigenesis and the prevention of colon cancer.

## Introduction

It is widely accepted that the accumulation of genetic and epigenetic alterations contributes to cancer initiation and progression. Genetic alterations refer to mutations in tumor suppressor genes and oncogenes, whereas epigenetic modifications involve changes in chromatin structure that result in altered gene expression without primary changes to the DNA sequence [1]. The information conveyed by epigenetic modifications plays a vital role in regulating DNA-mediated processes, including transcription, DNA repair, and replication [2]. Specifically, aberrant DNA methylation at the 5-carbon on cytosine residues (5mC) in CpG dinucleotides is perhaps the most extensively characterized epigenetic modification in cancer. DNA methylation affects the rate of gene transcription and therefore regulates various biological processes, such as proliferation, apoptosis, DNA repair, cancer initiation, and cancer progression [3]. The genomic DNA methylation pattern is stably maintained in normal cells; however, aberrant alterations in the epigenome have been identified in tumor cells [4]. Evidence suggests that global hypomethylation and regional hypermethylation are characteristics of cancer cells [5]. Global genome-wide loss of methylation has been associated with increased genomic instability and proto-oncogene activation, whereas DNA hypermethylation of CpG islands in promoter regions silences tumor suppressor genes [6]. Unlike genetic mutations, the transcriptional repression of genes via epigenetic alterations can be reversed by further epigenetic modifications because these silenced genes remain genetically intact [7]. Thus, it is very important to profile the global DNA methylation changes that occur in early tumorigenesis.

Colorectal cancer (CRC) is the second leading cause of cancer-related death in western countries [8], and more than 80 % of CRC patients harbor a mutation in the adenomatous polyposis coli (APC) gene on chromosome 5q21 [9]. APC is a tumor suppressor gene that down-regulates the pro-proliferative Wnt-signaling pathway by promoting the destruction of β-catenin. Deleterious mutations in APC stabilize β-catenin, increase its translocation into the nucleus, promote its binding to the transcription factor TCF4, and activate target genes such as C-MYC and CCND1 [10, 11]. It has been suggested that the loss of APC function initiates tumorigenesis and that additional genetic and epigenetic events are involved in colon cancer progression [12]. Numerous genes that are silenced by epigenetic mechanisms have been identified in colon cancer, including CDKN2A [13], DKK1 [14], DLEC1 [15, 16], UNC5C [17], and SFRP [18]. However, the genome-wide profile of the aberrant methylation and the association of these methylation patterns with important signaling pathways and biological networks implicated in colon tumorigenesis remain unclear.

To address this issue, we examined the global DNA methylation profile in the well-established Apc^min/+^ intestinal tumorigenesis mouse model using methylated DNA immunoprecipitation (MeDIP) and next-generation sequencing (MeDIP-seq). Apc^min/+^ mice carry a heterozygous mutation in Apc and develop approximately 30 small intestinal adenomatous polyps following the somatic loss of functional Apc [19]. This mouse model of intestinal tumorigenesis is commonly used because the phenotype resembles that of patients with familial adenomatous polyposis (FAP) [20]. We analyzed adenomatous polyps from Apc^min/+^ mice and not only identified genes with a modified methylation profile but also interpreted the data in the context of biological function, networks, and canonical signaling pathways associated with the methylation patterns.

## Results

### MeDIP-seq results

**Fig. 1:**
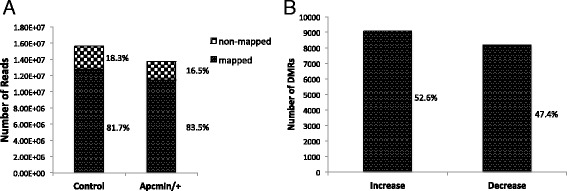
Global changes in the DNA methylation profile between Apc mutant adenomatous polyps and control tissue. **a**, Total number of peaks generated by MeDIP-seq. **b**, Number of DMRs with significantly increased or decreased changes in methylation (≥2-fold in log_2_) in polyps from Apc^min/+^ mice

To identify changes in DNA methylation patterns during the progression of mouse intestinal polyps, whole-genome DNA methylation analysis was performed using the described MeDIP-seq method. The global differences in the DNA methylation profile between adenomatous polyps from Apc^min/+^ mice and intestinal tissue from control mice are described in Fig. 1. We identified 12,761,009 mapped peaks and 2,868,549 non-mapped peaks from a total of 15,629,558 peaks in control mice and 11,470,541 mapped peaks and 2,262,073 non-mapped peaks from a total of 13,732,614 peaks in Apc^min/+^ mice (Fig. 1a). A total of 17,265 differentially methylated regions (DMRs) had a ≥ 2-fold change (log_2_) in methylation in Apc^min/+^ mice compared with control mice, of which 9,078 DMRs (52.6 %) exhibited increased methylation, and 8,187 (47.4 %) DMRs exhibited decreased methylation (Fig. 1b).

### Functional and pathway analysis by IPA

**Fig. 2:**
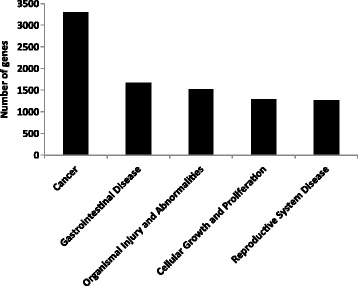
The 5 most significant biological functions and diseases related to changes in the methylation patterns. The number of molecules in the dataset associated with a known function was determined by IPA functional analysis

**Fig. 3:**
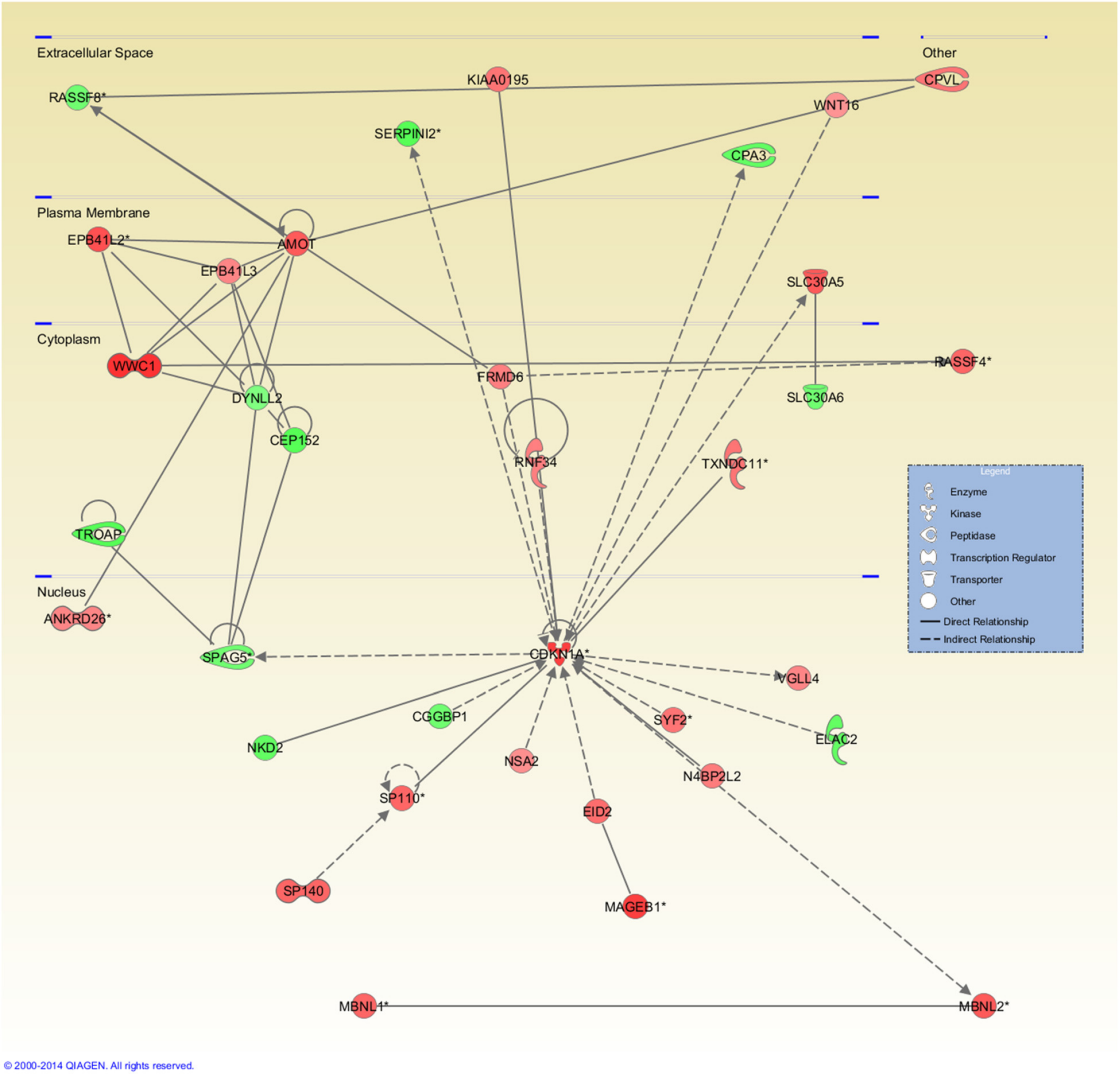
The most significant networks determined by IPA: cancer, cell cycle, and molecular transport. The IPA network analysis was conducted using the genes that were differentially methylated and their close relationships. IPA used triangle connectivity based on 30 focus genes and built the network according to the number of interactions between a single gene and others in the existing network. Red, increased methylation; green, decreased methylation

To identify the biological function, networks, and canonical pathways that were affected by the differentially methylated genes, we performed Ingenuity Pathway Analysis (IPA) after the MeDIP-seq analysis. In the analysis of genes with altered methylation (≥2-fold in log_2_) in Apc^min/+^ mice compared with control mice as determined by MeDIP-seq, IPA mapped 5,464 unique genes that were associated with its knowledge base. The top 50 genes with increased and decreased methylation levels based on log_2_ fold change are listed in Tables 1 and 2. The molecules with methylation changes were mainly categorized into 38 disease and biological functions. The five highest IPA-rated disease and biological functions were as follows: cancer, gastrointestinal disease, organismal injury and abnormalities, cellular growth and proliferation, and reproductive system disease (Fig. 2). Among the IPA-mapped genes with differential methylation patterns in polyps from Apc^min/+^ mice, 3,299 were associated with cancer, and 1,668 were associated with gastrointestinal diseases. To examine the interaction networks that were affected by DNA methylation in Apc mutant polyps, IPA identified 25 networks with up to 35 focus molecules in each network. The five most affected gene networks as determined by IPA are shown in Table 3, and the detailed interactions in the most significant networks (cancer, cell cycle, and molecular transport) are presented in Fig. 3. In accordance with the most relevant biological functions as determined by IPA, genes with different methylation patterns predominantly mapped to the networks associated with cancer and gastrointestinal diseases. Taken together, these results suggested an important role for the altered methylation of genes associated with the development of cancer and gut disease in Apc^min/+^ mice.

**Table 1:** Top 50 annotated genes with increased methylation

Rank	Symbol	Gene name	log_2_ Fold Change	Location	Type(s)
1	ZNF330	zinc finger protein 330	4.614	Nucleus	other
2	ACTR3B	ARP3 actin-related protein 3 homolog B (yeast)	4.540	Other	other
3	CAV3	caveolin 3	4.292	Plasma Membrane	enzyme
4	NKX2-3	NK2 homeobox 3	4.199	Nucleus	transcription regulator
5	TLN2	talin 2	4.199	Nucleus	other
6	CPD	carboxypeptidase D	4.100	Extracellular Space	peptidase
7	CTNNBL1	catenin, beta like 1	4.100	Nucleus	other
8	Vmn2r1	vomeronasal 2, receptor 1	4.100	Plasma Membrane	other
9	Cmtm2a	CKLF-like MARVEL transmembrane domain containing 2A	3.993	Cytoplasm	transcription regulator
10	HPS6	Hermansky-Pudlak syndrome 6	3.993	Cytoplasm	other
11	KANK1	KN motif and ankyrin repeat domains 1	3.993	Nucleus	transcription regulator
12	RRP1	ribosomal RNA processing 1	3.993	Nucleus	other
13	SNX10	sorting nexin 10	3.993	Cytoplasm	transporter
14	UNC93A	unc-93 homolog A (C. elegans)	3.993	Plasma Membrane	other
15	Zfp932	zinc finger protein 932	3.993	Nucleus	other
16	ANKRD13D	ankyrin repeat domain 13 family, member D	3.877	Plasma Membrane	other
17	DACT1	dishevelled-binding antagonist of beta-catenin 1	3.877	Cytoplasm	other
18	DMRT2	doublesex and mab-3 related transcription factor 2	3.877	Nucleus	other
19	DSC3	desmocollin 3	3.877	Plasma Membrane	other
20	LDOC1	leucine zipper, down-regulated in cancer 1	3.877	Nucleus	other
21	LRRC8B	leucine rich repeat containing 8 family, member B	3.877	Other	other
22	SEPP1	selenoprotein P, plasma, 1	3.877	Extracellular Space	other
23	SMAD3	SMAD family member 3	3.877	Nucleus	transcription regulator
24	Smok2a	sperm motility kinase 2B	3.877	Other	other
25	TCEAL3	transcription elongation factor A (SII)-like 3	3.877	Other	other
26	TNS1	tensin 1	3.877	Plasma Membrane	other
27	TRHR	thyrotropin-releasing hormone receptor	3.877	Plasma Membrane	G-protein coupled receptor
28	WWC1	WW and C2 domain containing 1	3.877	Cytoplasm	transcription regulator
29	PER2	period circadian clock 2	3.853	Nucleus	other
30	BHLHE23	basic helix-loop-helix family, member e23	3.752	Nucleus	transcription regulator
31	GALNT13	UDP-N-acetyl-alpha-D-galactosamine:polypeptide N-acetylgalactosaminyltransferase 13 (GalNAc-T13)	3.752	Cytoplasm	enzyme
32	KCNF1	potassium voltage-gated channel, subfamily F, member 1	3.752	Plasma Membrane	ion channel
33	MPP1	membrane protein, palmitoylated 1, 55 kDa	3.752	Plasma Membrane	kinase
34	OPA1	optic atrophy 1 (autosomal dominant)	3.752	Cytoplasm	enzyme
35	PTP4A1	protein tyrosine phosphatase type IVA, member 1	3.752	Cytoplasm	phosphatase
36	SGCZ	sarcoglycan, zeta	3.752	Plasma Membrane	other
37	ADCY7	adenylate cyclase 7	3.614	Plasma Membrane	enzyme
38	ALCAM	activated leukocyte cell adhesion molecule	3.614	Plasma Membrane	other
39	AR	androgen receptor	3.614	Nucleus	ligand-dependent nuclear receptor
40	C4orf33	chromosome 4 open reading frame 33	3.614	Other	other
41	CCNH	cyclin H	3.614	Nucleus	transcription regulator
42	CDKN1A	cyclin-dependent kinase inhibitor 1A (p21, Cip1)	3.614	Nucleus	kinase
43	CDV3	CDV3 homolog (mouse)	3.614	Cytoplasm	other
44	COMT	catechol-O-methyltransferase	3.614	Cytoplasm	enzyme
45	CRYGC	crystallin, gamma C	3.614	Cytoplasm	other
46	FAM13A	family with sequence similarity 13, member A	3.614	Cytoplasm	other
47	IGF1R	insulin-like growth factor 1 receptor	3.614	Plasma Membrane	transmembrane receptor
48	IYD	iodotyrosine deiodinase	3.614	Plasma Membrane	enzyme
49	JAG1	jagged 1	3.614	Extracellular Space	growth factor
50	KCNMA1	potassium large conductance calcium-activated channel, subfamily M, alpha member 1	3.614	Plasma Membrane	ion channel

**Table 2:** Top 50 annotated genes with decreased methylation

Rank	Symbol	Gene name	log_2_ Fold Change	Location	Type(s)
1	IRX1	iroquois homeobox 1	−5.897	Nucleus	transcription regulator
2	OSBP2	oxysterol binding protein 2	−5.408	Cytoplasm	other
3	CAPN5	calpain 5	−5.231	Cytoplasm	peptidase
4	INTS9	integrator complex subunit 9	−4.837	Nucleus	other
5	TRIML1	tripartite motif family-like 1	−4.837	Other	other
6	CSMD1	CUB and Sushi multiple domains 1	−4.614	Plasma Membrane	other
7	NCOR2	nuclear receptor corepressor 2	−4.272	Nucleus	transcription regulator
8	C6orf89	chromosome 6 open reading frame 89	−4.167	Other	other
9	TMEM242	transmembrane protein 242	−4.167	Other	other
10	DCLRE1A	DNA cross-link repair 1A	−4.100	Nucleus	other
11	EDNRA	endothelin receptor type A	−3.877	Plasma Membrane	transmembrane receptor
12	GALNT11	UDP-N-acetyl-alpha-D-galactosamine:polypeptide N-acetylgalactosaminyltransferase 11 (GalNAc-T11)	−3.877	Cytoplasm	enzyme
13	PTPN11	protein tyrosine phosphatase, non-receptor type 11	−3.877	Cytoplasm	phosphatase
14	AGPAT9	1-acylglycerol-3-phosphate O-acyltransferase 9	−3.795	Cytoplasm	enzyme
15	IER5	immediate early response 5	−3.795	Other	other
16	PPM1D	protein phosphatase, Mg2+/Mn2+ dependent, 1D	−3.708	Cytoplasm	phosphatase
17	RBBP6	retinoblastoma binding protein 6	−3.708	Nucleus	enzyme
18	BLOC1S2	biogenesis of lysosomal organelles complex-1, subunit 2	−3.614	Cytoplasm	other
19	CPEB2	cytoplasmic polyadenylation element binding protein 2	−3.614	Cytoplasm	other
20	ECI2	enoyl-CoA delta isomerase 2	−3.614	Cytoplasm	enzyme
21	MMGT1	membrane magnesium transporter 1	−3.614	Cytoplasm	transporter
22	NALCN	sodium leak channel, non-selective	−3.614	Plasma Membrane	ion channel
23	RETNLB	resistin like beta	−3.614	Extracellular Space	other
24	AMD1	adenosylmethionine decarboxylase 1	−3.515	Cytoplasm	enzyme
25	C1orf198	chromosome 1 open reading frame 198	−3.515	Other	other
26	DGKI	diacylglycerol kinase, iota	−3.515	Cytoplasm	kinase
27	DYNLT3	dynein, light chain, Tctex-type 3	−3.515	Cytoplasm	other
28	EPHA6	EPH receptor A6	−3.515	Plasma Membrane	kinase
29	GABRA6	gamma-aminobutyric acid (GABA) A receptor, alpha 6	−3.515	Plasma Membrane	ion channel
30	Gk2	glycerol kinase 2	−3.515	Cytoplasm	other
31	GLT1D1	glycosyltransferase 1 domain containing 1	−3.515	Extracellular Space	enzyme
32	HMGN2	high mobility group nucleosomal binding domain 2	−3.515	Nucleus	other
33	KLHL17	kelch-like family member 17	−3.515	Cytoplasm	other
34	Olfr266	olfactory receptor 266	−3.515	Plasma Membrane	G-protein coupled receptor
35	Ott	ovary testis transcribed	−3.515	Other	other
36	P2RX7	purinergic receptor P2X, ligand-gated ion channel, 7	−3.515	Plasma Membrane	ion channel
37	PTER	phosphotriesterase related	−3.515	Other	enzyme
38	Rnf213	ring finger protein 213	−3.515	Cytoplasm	enzyme
39	SERPINC1	serpin peptidase inhibitor, clade C (antithrombin), member 1	−3.515	Extracellular Space	enzyme
40	TPD52L1	tumor protein D52-like 1	−3.515	Cytoplasm	other
41	ZMAT4	zinc finger, matrin-type 4	−3.515	Nucleus	other
42	RBM20	RNA binding motif protein 20	−3.462	Nucleus	other
43	BEGAIN	brain-enriched guanylate kinase-associated	−3.408	Nucleus	other
44	CHSY3	chondroitin sulfate synthase 3	−3.408	Cytoplasm	enzyme
45	CKAP4	cytoskeleton-associated protein 4	−3.408	Cytoplasm	other
46	DPF3	D4, zinc and double PHD fingers, family 3	−3.408	Other	other
47	Ear2	eosinophil-associated, ribonuclease A family, member 2	−3.408	Cytoplasm	enzyme
48	FAM135B	family with sequence similarity 135, member B	−3.408	Other	enzyme
49	POT1	protection of telomeres 1	−3.408	Nucleus	other
50	POU6F1	POU class 6 homeobox 1	−3.408	Nucleus	transcription regulator

**Table 3:** Ingenuity Pathway Analysis of gene networks

Rank	Molecules in network	Score	Focus molecules	Top function
1	↑AMOT,↑ANKRD26,↑CDKN1A,↓CEP152,↓CGGBP1,↓CPA3,↑CPVL, ↓DYNLL2, ↑EID2, ↓ELAC2, ↑EPB41L2, ↑EPB41L3, ↑FRMD6, ↑KIAA0195, ↑MAGEB1, ↑MBNL1, ↑MBNL2, ↑N4BP2L2, ↓NKD2, ↑NSA2, ↑RASSF4, ↓RASSF8, ↑RNF34, ↓SERPINI2, ↑SLC30A5, ↓SLC30A6, ↑SP110, ↑SP140, ↓SPAG5, ↑SYF2, ↓TROAP, ↑TXNDC11, ↑VGLL4, ↑WNT16, ↑WWC1	30	35	Cancer, Cell Cycle, Molecular Transport
2	↑ACACA, ↓ATRNL1, ↓BHMT, ↑CYP2A13, ↓Cyp2c70, ↑CYP3A43, ↓DCLRE1A, ↑E330013P04Rik, ↓FASN, ↑GPC6, ↑GSTP1, ↓HNMT, ↓IVNS1ABP, ↓Keg1, ↓Lcn4, ↑LRTM1, ↓MC4R, ↓Mill1, ↑MRGPRX3, ↑MT1E, ↑MTF1, ↑NR1H4, ↑RORA, ↑SLC13A1, ↑SLC16A7, ↓SLC29A4, ↓SLC30A1, ↓SLC38A4, ↑SULT1C3, ↓TMC6, ↓UCP1, ↑UPP2, ↓Xlr3c (includes others), ↑ZNF275, ↓ZNF292	30	35	Renal Damage, Renal Tubule Injury, Molecular Transport
3	↑ABTB2, ↑ALKBH8, ↑ALPK1, ↓BCKDHB, ↑BTBD7, ↑C11orf70, ↑C20orf194, ↑CAMKV, ↓CCDC39, ↑CUL2, ↓CUL3, ↑DCLK2, ↑EGFLAM, ↑FAM98A, ↓FARS2, ↑FBXO10, ↑FBXO34, ↑G2E3, ↓G3BP2, ↑HSP90AA1, ↓KCNG1, ↓KCNS3, ↓KCTD8, ↑KLHL10, ↓KLHL14, ↑KLHL29, ↑KLHL32, ↑KLHL36, ↑KRR1, ↑QDPR, ↑RCBTB1, ↓SEPHS1, ↓UST, ↓YWHAE, ↑ZBED4	30	35	Hereditary Disorder, Respiratory Disease, Metabolic Disease
4	↓ABCA6, ↓ABLIM3, ↓ABRA, ↑AIF1L, ↓AMBRA1, ↓ARAP2, ↓ARL6, ↓ATL2, ↓CAPN5, ↑CAPN6, ↓CASP12,CD80/CD86, ↑CLEC2D, ↑CLEC6A, ↑CRTAM, ↑GBP5, ↑Gbp8,Gbp6 (includes others), ↑GFM1, ↑GIMAP1-GIMAP5, ↑Gvin1 (includes others), ↑HERC6, ↑IFNG, ↓KIAA0226, ↓KIF16B,↑KLRB1, ↓KMO, ↓KY, ↑LAMP3, ↑LIX1, ↓Neurl3, ↑PCDH17, ↑Phb, ↑PILRB, ↓PMP2	28	34	Endocrine System Disorders, Gastrointestinal Disease, Immunological Disease
5	↑AFF2, ↑AP4S1, ↑ASAP2, ↓C21orf91, ↑C2orf88, ↑DLGAP1, ↑Eif2s3x, ↓FAM110A, ↑GNS, ↑GRB2, ↑HDGFRP3, ↓KCNH7, ↓KIRREL, ↑KRT83, ↑LRFN4, ↑MEPE, ↑NCK1, ↑NCKAP5, ↓PANX2, ↑PHACTR2, ↑RALGAPA2, ↓RALGPS1, ↑SEPN1, ↑SH2D4A, ↑SHANK2, ↓SHROOM2, ↓SLCO2A1, ↑SNX8, ↑SNX12, ↑SNX18, ↓SPRY, ↑TJAP1, ↑TTYH2, ↑WDR44, ↑ZNF32	28	34	Cellular Assembly and Organization, Tissue Development, Cellular Function and Maintenance

↑, increased methylation; ↓, decreased methylation

**Fig. 4:**
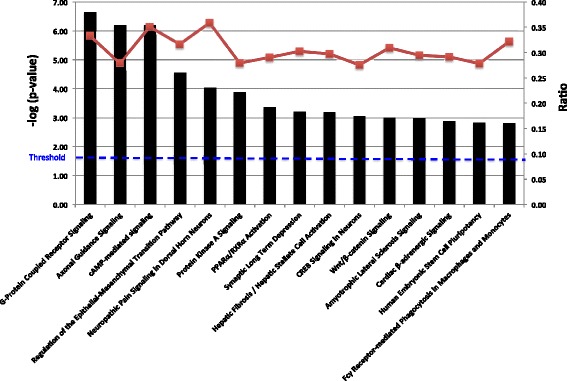
The 15 most significant canonical pathways related to changes in the methylation patterns. The left y-axis (bar graph) presents the data as the log (*p*-value) of each pathway using Fisher’s exact test. The right y-axis (line graph) corresponds to the ratio data for each pathway. The ratios were calculated as the number of input molecules mapped to a specific pathway divided by the total number of molecules in the given pathway

**Fig. 5:**
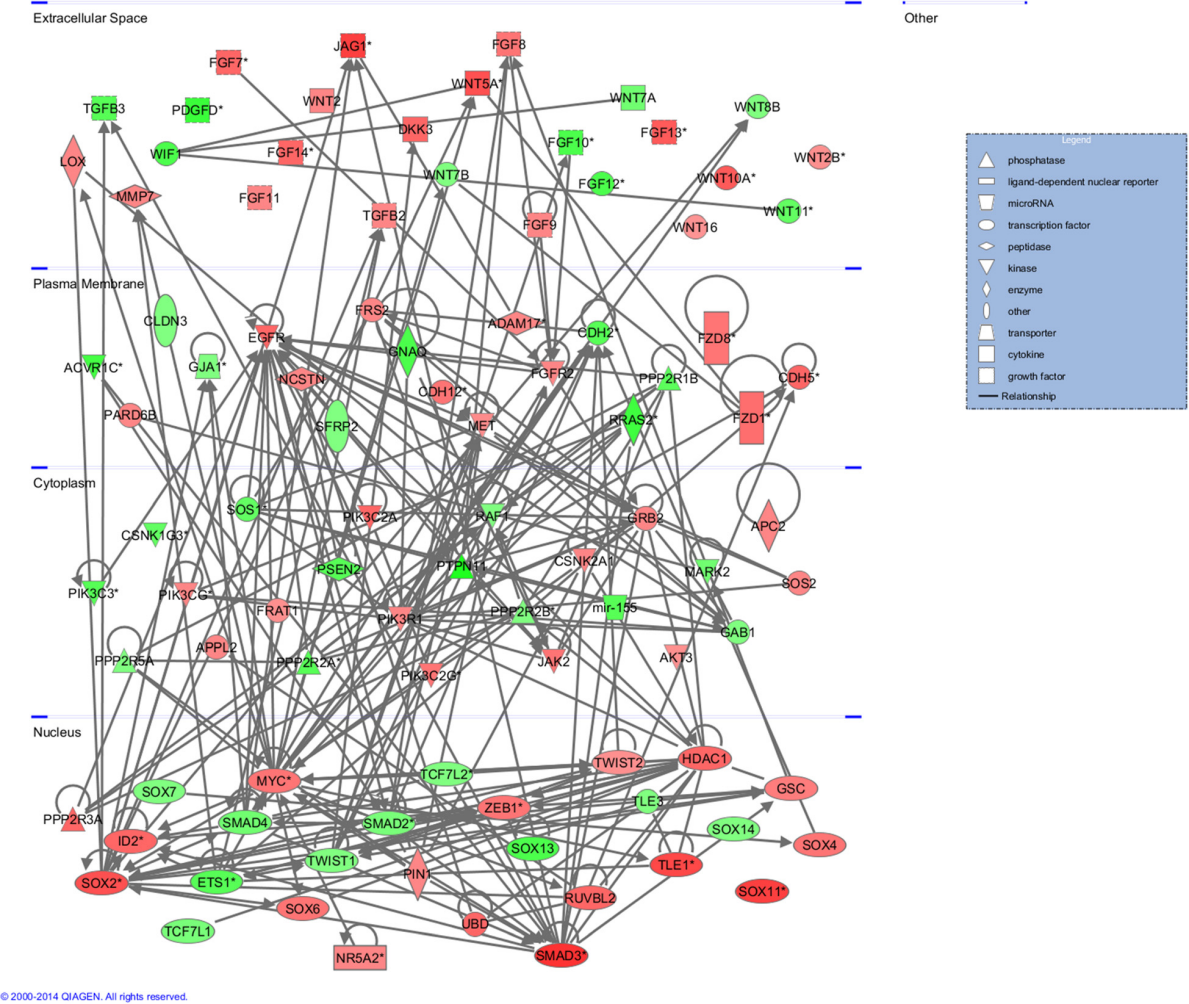
Predicted interactions between molecules with altered methylation that mapped to the EMT and Wnt/β-catenin pathways. IPA predicted direct interaction of the genes with altered methylation patterns in the EMT and Wnt/β-catenin pathways based on the publication database. Red, increased methylation; green, decreased methylation

Canonical pathways associated with methylation changes in Apc mutant polyps were analyzed based on the ratio of the number of input genes to the total number of reference genes in the corresponding pathways in the IPA knowledge bases. Fisher’s exact test was employed to calculate the P values to determine whether the associations between the differentially methylated genes and the canonical pathways were significant or random. Using a cutoff value of P < 0.05, IPA identified 84 significant signaling pathways that contained genes with increased or decreased methylation. The 15 most significant pathways that correlated with methylation changes in polyps are presented in Fig. 4. Notably, regulation of the epithelial-mesenchymal transition (EMT) pathway was mapped by IPA and ranked as the 4th most significant canonical pathway associated with altered methylation. According to the IPA knowledge bases, the regulation of the EMT pathway includes 196 molecules. Among these molecules, 62 displayed greater than a 2 fold change (log_2_) in methylation in the polyps from Apc^min/+^ mice by MeDIP-seq. The abnormal methylation changes in the EMT pathway included alterations in the methylation profiles of kinases, peptidases, phosphatases, transcription regulators, transmembrane receptors, and microRNAs. Tables 4 and 5 lists the genes involved in the EMT pathway that exhibited altered methylation (37 genes with increased methylation in Table 4; 25 genes with decreased methylation in Table 5). Signaling pathways, such as the Wnt/β-catenin, TGF-β, NOTCH, and receptor tyrosine kinase (RTK) pathways, can initiate an EMT program alone or in combination [21]. Although the genes that were determined to have differential methylation patterns in polyps by MeDIP-seq were not significantly associated with the TGF-β, NOTCH, and RTK signaling pathways, the Wnt/β-catenin pathway was identified as one of the most significant canonical pathways implicated based on methylation changes in the polyps (ranked 11th). Specifically, 53 out of 175 molecules in this pathway showed methylation changes of greater than 2-fold (log_2_) in polyps from Apc^min/+^ mice; these molecules are listed in Tables 6 and 7 (30 genes with increased methylation in Table 6; 23 genes with decreased methylation in Table 7). Additionally, we found many shared genes in the EMT and Wnt/β-catenin pathways with altered methylation levels; these genes are shown in bold in Tables 4, 5, 6 and 7. To understand the role of DNA methylation in the crosstalk between the EMT and Wnt/β-catenin pathways in Apc^min/+^ mice, IPA was utilized to predict the direct interaction of the differentially methylated genes in these two pathways based on the publication database (Fig. 5). The pathway analysis of the MeDIP-seq data suggested that cellular changes mediated via the EMT and Wnt/β-catenin pathways may be significantly associated with altered DNA methylation in polyps from Apc^min/+^ mice.

**Table 4:** Genes with increased methylation that mapped to the regulation of the EMT pathway by IPA

Symbol	Gene name	log_2_ Fold Change	Location	Type(s)
SMAD3	SMAD family member 3	3.877	Nucleus	transcription regulator
JAG1	jagged 1	3.614	Extracellular Space	growth factor
**WNT5A**	**wingless-type MMTV integration site family, member 5A**	**3.292**	**Extracellular Space**	**cytokine**
FGF13	fibroblast growth factor 13	3.100	Extracellular Space	growth factor
**WNT10A**	**wingless-type MMTV integration site family, member 10A**	**3.100**	**Extracellular Space**	**other**
EGFR	epidermal growth factor receptor	2.877	Plasma Membrane	kinase
FGF7	fibroblast growth factor 7	2.877	Extracellular Space	growth factor
FGF14	fibroblast growth factor 14	2.877	Extracellular Space	growth factor
ID2	inhibitor of DNA binding 2, dominant negative helix-loop-helix protein	2.877	Nucleus	transcription regulator
PIK3C2A	phosphatidylinositol-4-phosphate 3-kinase, catalytic subunit type 2 alpha	2.877	Cytoplasm	kinase
**FZD1**	**frizzled class receptor 1**	**2.752**	**Plasma Membrane**	**G-protein coupled receptor**
**CDH12**	**cadherin 12, type 2 (N-cadherin 2)**	**2.614**	**Plasma Membrane**	**other**
FGF8	fibroblast growth factor 8 (androgen-induced)	2.614	Extracellular Space	growth factor
**FZD8**	**frizzled class receptor 8**	**2.614**	**Plasma Membrane**	**G-protein coupled receptor**
JAK2	Janus kinase 2	2.614	Cytoplasm	kinase
PIK3C2G	phosphatidylinositol-4-phosphate 3-kinase, catalytic subunit type 2 gamma	2.614	Cytoplasm	kinase
ZEB1	zinc finger E-box binding homeobox 1	2.614	Nucleus	transcription regulator
GSC	goosecoid homeobox	2.462	Nucleus	transcription regulator
ADAM17	ADAM metallopeptidase domain 17	2.292	Plasma Membrane	peptidase
FGF9	fibroblast growth factor 9	2.292	Extracellular Space	growth factor
FGF11	fibroblast growth factor 11	2.292	Extracellular Space	growth factor
FGFR2	fibroblast growth factor receptor 2	2.292	Plasma Membrane	kinase
FRS2	fibroblast growth factor receptor substrate 2	2.292	Plasma Membrane	other
GRB2	growth factor receptor-bound protein 2	2.292	Cytoplasm	other
LOX	lysyl oxidase	2.292	Extracellular Space	enzyme
NCSTN	nicastrin	2.292	Plasma Membrane	peptidase
PARD6B	par-6 family cell polarity regulator beta	2.292	Plasma Membrane	other
PIK3CG	phosphatidylinositol-4,5-bisphosphate 3-kinase, catalytic subunit gamma	2.292	Cytoplasm	kinase
PIK3R1	phosphoinositide-3-kinase, regulatory subunit 1 (alpha)	2.292	Cytoplasm	kinase
SOS2	son of sevenless homolog 2 (Drosophila)	2.292	Cytoplasm	other
**TGFB2**	**transforming growth factor, beta 2**	**2.292**	**Extracellular Space**	**growth factor**
**WNT2**	**wingless-type MMTV integration site family member 2**	**2.292**	**Extracellular Space**	**cytokine**
MET	MET proto-oncogene, receptor tyrosine kinase	2.180	Plasma Membrane	kinase
**AKT3**	**v-akt murine thymoma viral oncogene homolog 3**	**2.100**	**Cytoplasm**	**kinase**
TWIST2	twist family bHLH transcription factor 2	2.100	Nucleus	transcription regulator
**WNT2B**	**wingless-type MMTV integration site family, member 2B**	**2.100**	**Extracellular Space**	**other**
**WNT16**	**wingless-type MMTV integration site family, member 16**	**2.029**	**Extracellular Space**	**other**

**Table 5:** Genes with decreased methylation that mapped to the regulation of the EMT pathway by IPA

Symbol	Gene name	log_2_ Fold Change	Location	Type(s)
PTPN11	protein tyrosine phosphatase, non-receptor type 11	−3.877	Cytoplasm	phosphatase
PDGFD	platelet derived growth factor D	−3.167	Extracellular Space	growth factor
RRAS2	related RAS viral (r-ras) oncogene homolog 2	−3.090	Plasma Membrane	enzyme
FGF10	fibroblast growth factor 10	−2.877	Extracellular Space	growth factor
FGF12	fibroblast growth factor 12	−2.877	Extracellular Space	other
**CDH2**	**cadherin 2, type 1, N-cadherin (neuronal)**	**−2.708**	**Plasma Membrane**	**other**
ETS1	v-ets avian erythroblastosis virus E26 oncogene homolog 1	−2.708	Nucleus	transcription regulator
mir-155	microRNA 155	−2.708	Cytoplasm	microRNA
PIK3C3	phosphatidylinositol 3-kinase, catalytic subunit type 3	−2.708	Cytoplasm	growth factor
PSEN2	presenilin 2	−2.708	Cytoplasm	peptidase
**TGFB3**	**transforming growth factor, beta 3**	**−2.708**	**Extracellular Space**	**growth factor**
SOS1	son of sevenless homolog 1 (Drosophila)	−2.515	Cytoplasm	other
**WNT11**	**wingless-type MMTV integration site family, member 11**	**−2.515**	**Extracellular Space**	**other**
SMAD4	SMAD family member 4	−2.292	Nucleus	transcription regulator
**WNT7A**	**wingless-type MMTV integration site family, member 7A**	**−2.292**	**Extracellular Space**	**cytokine**
SMAD2	SMAD family member 2	−2.167	Nucleus	transcription regulator
**TCF7L1**	**transcription factor 7-like 1 (T-cell specific, HMG-box)**	**−2.167**	**Nucleus**	**transcription regulator**
CLDN3	claudin 3	−2.029	Plasma Membrane	transmembrane receptor
GAB1	GRB2-associated binding protein 1	−2.029	Cytoplasm	other
HMGA2	--	−2.029	Other	other
RAF1	Raf-1 proto-oncogene, serine/threonine kinase	−2.029	Cytoplasm	kinase
**TCF7L2**	**transcription factor 7-like 2 (T-cell specific, HMG-box)**	**−2.029**	**Nucleus**	**transcription regulator**
TWIST1	twist family bHLH transcription factor 1	−2.029	Nucleus	transcription regulator
**WNT7B**	**wingless-type MMTV integration site family, member 7B**	**−2.029**	**Extracellular Space**	**other**
**WNT8B**	**wingless-type MMTV integration site family, member 8B**	**−2.029**	**Extracellular Space**	**other**

**Table 6:** Genes with increased methylation that mapped to the Wnt/β-catenin pathway by IPA

Symbol	Gene name	log_2_ Fold Change	Location	Type(s)
SOX11	SRY (sex determining region Y)-box 11	3.614	Nucleus	transcription regulator
TLE1	transducin-like enhancer of split 1 (E(sp1) homolog, Drosophila)	3.462	Nucleus	transcription regulator
SOX2	SRY (sex determining region Y)-box 2	3.292	Nucleus	transcription regulator
**WNT5A**	**wingless-type MMTV integration site family, member 5A**	**3.292**	**Extracellular Space**	**cytokine**
**WNT10A**	**wingless-type MMTV integration site family, member 10A**	**3.100**	**Extracellular Space**	**other**
CDH5	cadherin 5, type 2 (vascular endothelium)	2.877	Plasma Membrane	other
DKK3	dickkopf WNT signaling pathway inhibitor 3	2.877	Extracellular Space	cytokine
HDAC1	histone deacetylase 1	2.877	Nucleus	transcription regulator
PPP2R3A	protein phosphatase 2, regulatory subunit B”, alpha	2.877	Nucleus	phosphatase
RUVBL2	RuvB-like AAA ATPase 2	2.877	Nucleus	transcription regulator
UBD	ubiquitin D	2.877	Nucleus	other
**FZD1**	**frizzled class receptor 1**	**2.752**	**Plasma Membrane**	**G-protein coupled receptor**
**CDH12**	**cadherin 12, type 2 (N-cadherin 2)**	**2.614**	**Plasma Membrane**	**other**
**FZD8**	**frizzled class receptor 8**	**2.614**	**Plasma Membrane**	**G-protein coupled receptor**
MYC	v-myc avian myelocytomatosis viral oncogene homolog	2.614	Nucleus	transcription regulator
SOX4	SRY (sex determining region Y)-box 4	2.614	Nucleus	transcription regulator
SOX6	SRY (sex determining region Y)-box 6	2.614	Nucleus	transcription regulator
APC2	adenomatosis polyposis coli 2	2.292	Cytoplasm	enzyme
APPL2	adaptor protein, phosphotyrosine interaction, PH domain and leucine zipper containing 2	2.292	Cytoplasm	other
CSNK2A1	casein kinase 2, alpha 1 polypeptide	2.292	Cytoplasm	kinase
MMP7	matrix metallopeptidase 7 (matrilysin, uterine)	2.292	Extracellular Space	peptidase
NR5A2	nuclear receptor subfamily 5, group A, member 2	2.292	Nucleus	ligand-dependent nuclear receptor
PIN1	peptidylprolyl cis/trans isomerase, NIMA-interacting 1	2.292	Nucleus	enzyme
TGFB2	transforming growth factor, beta 2	2.292	Extracellular Space	growth factor
**WNT2**	**wingless-type MMTV integration site family member 2**	**2.292**	**Extracellular Space**	**cytokine**
**AKT3**	**v-akt murine thymoma viral oncogene homolog 3**	**2.100**	**Cytoplasm**	**kinase**
FRAT1	frequently rearranged in advanced T-cell lymphomas	2.100	Cytoplasm	other
**WNT2B**	**wingless-type MMTV integration site family, member 2B**	**2.100**	**Extracellular Space**	**other**
**WNT16**	**wingless-type MMTV integration site family, member 16**	**2.029**	**Extracellular Space**	**other**

**Table 7:** Genes with decreased methylation that mapped to the Wnt/β-catenin pathway by IPA

Symbol	Gene name	log_2_ Fold Change	Location	Type(s)
ACVR1C	activin A receptor, type IC	−3.029	Plasma Membrane	kinase
GNAQ	guanine nucleotide binding protein (G protein), q polypeptide	−2.877	Plasma Membrane	enzyme
SOX13	SRY (sex determining region Y)-box 13	−2.877	Nucleus	transcription regulator
WIF1	WNT inhibitory factor 1	−2.877	Extracellular Space	other
**CDH2**	**cadherin 2, type 1, N-cadherin (neuronal)**	**−2.708**	**Plasma Membrane**	**other**
PPP2R2A	protein phosphatase 2, regulatory subunit B, alpha	−2.708	Cytoplasm	phosphatase
**TGFB3**	**transforming growth factor, beta 3**	**−2.708**	**Extracellular Space**	**growth factor**
PPP2R1B	protein phosphatase 2, regulatory subunit A, beta	−2.614	Plasma Membrane	phosphatase
CSNK1G3	casein kinase 1, gamma 3	−2.515	Cytoplasm	kinase
**WNT11**	**wingless-type MMTV integration site family, member 11**	**−2.515**	**Extracellular Space**	**other**
MARK2	MAP/microtubule affinity-regulating kinase 2	−2.292	Cytoplasm	kinase
**WNT7A**	**wingless-type MMTV integration site family, member 7A**	**−2.292**	**Extracellular Space**	**cytokine**
**TCF7L1**	**transcription factor 7-like 1 (T-cell specific, HMG-box)**	**−2.167**	**Nucleus**	**transcription regulator**
GJA1	gap junction protein, alpha 1, 43 kDa	−2.029	Plasma Membrane	transporter
PPP2R2B	protein phosphatase 2, regulatory subunit B, beta	−2.029	Cytoplasm	phosphatase
PPP2R5A	protein phosphatase 2, regulatory subunit B’, alpha	−2.029	Cytoplasm	phosphatase
SFRP2	secreted frizzled-related protein 2	−2.029	Plasma Membrane	transmembrane receptor
SOX7	SRY (sex determining region Y)-box 7	−2.029	Nucleus	transcription regulator
SOX14	SRY (sex determining region Y)-box 14	−2.029	Nucleus	transcription regulator
**TCF7L2**	**transcription factor 7-like 2 (T-cell specific, HMG-box)**	**−2.029**	**Nucleus**	**transcription regulator**
TLE3	transducin-like enhancer of split 3	−2.029	Nucleus	other
**WNT7B**	**wingless-type MMTV integration site family, member 7B**	**−2.029**	**Extracellular Space**	**other**
**WNT8B**	**wingless-type MMTV integration site family, member 8B**	**−2.029**	**Extracellular Space**	**other**

## Discussion

Global hypomethylation and hypermethylation of CpG islands in tumor suppressor genes occurs in human colon cancer cell lines and primary colon adenomatous tissues [12]. However, the global genomic distribution of aberrant methylation and the association of these methylation signatures with pivotal signaling pathways and biological networks in colon cancer remain unclear, mainly due to the limitations of the existing techniques for analyzing DNA methylation at specific sequences [22]. Recently, the development of the MeDIP-based approach has enabled the rapid and comprehensive identification of multiple CpG sites. MeDIP in conjunction with high-throughput sequence (MeDIP-seq) provides a genome-wide mapping technique that has been successfully used to profile the global DNA methylation patterns of many cancer models [23–26]. Notably, Grimm et al. used MeDIP-seq to identify a large number of DMRs with distinct methylation patterns in Apc mutant adenomas, which are partially conserved between intestinal adenomas in Apc^min/+^ mice and human colon cancer [27]. In the present study, we used pathway analysis after MeDIP-seq to screen the global genomic methylation profile to identify genomic loci with aberrant methylation patterns in adenomatous polyps from Apc^min/+^ mice and to determine the biological function, networks, and canonical pathways that were affected by the DNA methylation in Apc mutant adenomas.

The top-ranked genes with increased and decreased methylation may provide information to facilitate the discovery of key genes, therapeutic targets, and biomarkers for the development, diagnosis, prognosis, and prevention of colon cancer. For example, CTNNBL1 [catenin (cadherin-associated protein) b-like 1] exhibited increased methylation in adenomatous polyp tissue (log_2_ fold change = 4.1, Table 1), as evidenced by MeDIP-seq. The CTNNBL1 gene is associated with obesity, a known risk factor for the development of CRC [28]. Recently, CTNNBL1 was reported to be a putative regulator of the canonical Wnt signaling pathway, and mutations in and dysregulation of this pathway are involved in CRC [29]. However, the potential epigenetic regulation of CTNNBL1 in colon cancer remains to be elucidated. To the best of our knowledge, this is the first report to suggest that CTNNBL1 might by aberrantly methylated in Apc mutant mice. Further experiments are necessary to investigate the epigenetic regulation of CTNNBL1 in colon cancer cells and patient specimens. CDKN1A (cyclin-dependent kinase inhibitor 1A, p21) showed increased methylation (log_2_ fold change = 3.6, Table 1) in adenomatous polyp tissue compared with control tissue. CDKN1A is a cyclin-dependent kinase inhibitor that plays a key role in regulating the cell cycle, especially the G1/S checkpoint, and its expression is lost in most cases of colon cancer. By analyzing 737 CRC samples, Ogino et al. concluded that the down-regulation of p21 inversely correlates with microsatellite instability and the CpG island methylator phenotype in colon cancer [30]. Here, we provided additional evidence by demonstrating potentially increased p21 methylation in Apc^min/+^ polyps.

It is commonly believed that promoter hypermethylation is associated with silencing of tumor suppressor genes in carcinogenesis [31]. One study observed a significant increase in DNA methylation in primary colon adenocarcinoma samples relative to normal colon tissue by analyzing the DNA methylation data from Cancer Genome Atlas (TCGA) and found an inverse correlation between DNA methylation and gene expression: genes with cancer-specific DNA methylation showed decreased transcription activity in colon adenocarcinoma [32]. However, Grimm et al. reported that the correlation of gene expression and DNA methylation applies only to a small set of genes by analyzing the results from MeDIP-seq and RNA-seq in normal intestine tissues and Apc mutant adenomas. In addition, they analyzed the mRNA expression of 31 selected tumor suppressors, only 2 were found both promoter hypermethylated and transcriptionally silenced. Surprisingly, the majority of tumor suppressors examined in their study did not exhibit a decreased transcriptional activity in adenoma compared to normal intestine samples [27]. These results suggested that silencing of tumor suppressor genes by aberrant methylation may not be common events during early polyposis of Apc mutant mice. Nevertheless, it is possible that epigenetic changes mediated gene silencing arises during progression of adenoma to carcinoma [33]. Furthermore, it was reported that instead of directly intervene active promoters, DNA methylation affects genes that are already silent by other mechanisms such as histone modifications [34]. Thus, further studies are needed to elucidate the dynamic changes of DNA methylation, histone modifications, and gene transcription in different stages, such as initiation, progression, and metastasis during colon carcinogenesis.

This study aimed to discover functions and pathways associated with epigenomic alterations in colon cancer in addition to the individual affected molecules. We utilized IPA to interpret the MeDIP-seq data in the context of molecular interactions, networks, and canonical pathways. IPA revealed that the genes with altered methylation patterns in adenomatous tissues predominantly occupied the cancer and cell cycle networks (Table 3) and the cancer and gastrointestinal disease functional categories (Fig. 2). This information suggested that dynamic epigenetic modifications might occur in genes associated with cancer, cell cycle regulation, and gut disease development in Apc^min/+^ mice.

Biological changes that lead to the switch from an epithelial to a mesenchymal cell phenotype, defined as EMT, play an important role in embryonic development and carcinogenesis [35]. In the context of tumorigenesis-associated EMT, neoplastic cells lose epithelial characteristics, such as cell-cell adhesion, cell polarity, and lack of motility, and acquire mesenchymal features, such as migratory ability, invasiveness, plasticity, and resistance to apoptosis [21]. The morphological alterations that occur during EMT enable neoplastic cells to escape from the basement membrane, migrate to neighboring lymph nodes, and eventually enter the circulation to establish secondary colonies at distant sites [36]. Thus, EMT program activation is considered a critical step in tumor growth, angiogenesis, and metastasis [37]. Chen et al. reported elevated expression of the mesenchymal marker vimentin in intestinal adenomas from Apc^min/+^ mice and suggested that molecular alterations in the initial steps of EMT are involved in early tumorigenesis in Apc^min/+^ mice; the early stages of intestinal tumorigenesis lack signs of invasion and metastasis [38]. These interesting observations highlighted the necessity to study the EMT process during early tumorigenesis. Although the molecular and biochemical mechanisms involved in the initiation and regulation of EMT in carcinogenesis are not yet fully understood, they appear to be associated with growth factor receptors (for example, RTKs), signaling pathways (for example, the Wnt/β-catenin, NOTCH, and TGF-β pathways), and stimuli (for example, oxidative stress) [39]. The involvement of epigenetic events in regulating the EMT proteome during carcinogenesis was recently demonstrated [40]. Using ChIP-seq (chromatin immunoprecipitation followed by sequencing) assays, Cieslik et al. showed that EMT is driven by the chromatin-mediated activation of transcription factors [41]. The current study identified many genes with increased or decreased methylation in the EMT pathway (Fig. 3, Tables 4 and 5), suggesting that aberrant DNA methylation may be associated with the activation of EMT during tissue remodeling in early tumorigenesis in Apc^min/+^ mice. The present study also provided useful information regarding important molecules in the EMT pathway that undergo alterations in their methylation pattern during polyposis in Apc^min/+^ mice. For example, SMAD3 (mothers against decapentaplegic homolog 3), a molecule that plays an essential role in TGF-β pathway-mediated EMT, was one of the genes that exhibited increased methylation (log_2_ fold change = 3.9, Table 4) in adenomas in Apc^min/+^ mice. Interestingly, SMAD3 deficiency promotes tumor formation in the distal colon of Apc^min/+^ mice [42]. EGFR (epidermal growth factor receptor), another important molecule that exhibited increased methylation, has been implicated in EMT in adenomas (log_2_ fold change = 2.9, Table 4). EGFR can induce EMT in cancer cells by up-regulating Twist [43], and promoter methylation of EGFR has been detected in metastatic tumors from patients with CRC [44]. The results of the current study indicated that aberrant methylation of EGFR may occur during early tumorigenesis in Apc^min/+^ mice. Important transcription factors in the EMT pathway, including ZEB 1 and TWIST 2, also exhibited increased methylation in adenomas from Apc^min/+^ mice (Table 4). Although the contribution of TWIST 2 to promoting EMT in breast cancer progression was recently reported [45], there is limited knowledge of the role of TWIST 2 in colon cancer; however, one study proposed that TWIST 2 is a potential prognostic biomarker for colon cancer [46]. Notably, aberrant methylation of TWIST 2 has been demonstrated in chronic lymphocytic leukemia [47] and acute lymphoblastic leukemia [48]. The present study is the first to suggest that methylation of the TWIST 2 gene may be involved in tumorigenesis in Apc^min/+^ mice. Further studies are necessary to elucidate the role of DNA methylation in EMT pathway regulation in early tumorigenesis in Apc^min/+^ mice.

Apc^min/+^ mice are thought to have a hyperactive Wnt/β-catenin pathway [10], but the epigenetic modifications of the Wnt/β-catenin pathway are still not fully understood. IPA identified the Wnt/β-catenin pathway as one of the most significant canonical pathways that contained genes with increased or decreased methylation, suggesting an important role for epigenetic alterations in the Wnt/β-catenin pathway in tumorigenesis. Some of the molecules with increased or decreased methylation patterns that were mapped to this pathway in the present study are consistent with the findings of previous publications. For example, Dhir et al. analyzed tissue samples from inflammatory bowel disease (IBD) and colon cancer patients and demonstrated that aberrant methylation of Wnt/β-catenin signaling genes is an early event in IBD-associated colon cancer. Aberrant methylation of APC2 (adenomatousis polyposis coli 2), SFRP1 (secreted frizzled-related protein 1), and SFRP2 (secreted frizzled-related protein 2) is associated with the progression from colitis to neoplasia [49]. In the current study, we observed increased methylation of APC2 and decreased methylation of SFRP2 in adenomas in Apc^min/+^ mice (Tables 6 and 7). Wang et al. demonstrated that black raspberries can prevent colonic ulceration in a DSS-induced model and in interleukin-10 knockout mice by epigenetically modifying genes with hypermethylated promoters in the Wnt/β-catenin pathway, such as DKK3 (dickkopf-related protein 3), APC, SFRP1, and SOX17 [SRY (sex determining region Y)-box 17] [50, 51]. In the present study, DKK3 consistently displayed increased methylation (log_2_ fold change = 2.9, Table 6) in adenomas from Apc^min/+^ mice compared with normal tissue. Furthermore, we provided additional information regarding the genes with altered methylation in the Wnt/β-catenin pathway in polyps from Apc^min/+^ mice, potentially facilitating future research on the involvement of aberrantly methylated Wnt/β-catenin pathway components in colon cancer development and on potential targets for epigenetic modification for the prevention of colon cancer. Intestinal adenoma in mouse originated from intestinal stem cells (ISC), a small fraction of cells in proliferative crypts [52]. Interestingly, Grimm and co-workers demonstrated that the adenoma-specific methylation signatures are not acquired from ISC by showing that the methylation patterns were similar in ISC, proliferative crypt cells, and differentiated villus cells, but are distinct in adenoma tissue [27]. Since ISC are responsive to Wnt signaling and we identified Wnt/β-catenin pathway as one of the most significant pathways associated with DNA methylation in polyps from Apc^min/+^ mice, it would be important to understand the mechanisms underlying the acquisition of aberrant DNA methylation patterns in Wnt/β-catenin pathway in adenoma and how the hypermethylated genes involved in Wnt/β-catenin pathway influence the neoplastic transformation from ISC to adenoma. Furthermore, the Wnt/β-catenin pathway is intimately associated with EMT pathway [53]. The present study provided valuable information regarding the potential crosstalk between the EMT and Wnt/β-catenin pathways, which are both affected by DNA methylation in Apc^min/+^ mice (Fig. 5). Further studies are needed to understand the role of the complex crosstalk between multiple signaling pathways in the progression of colon cancer.

In addition to DNA methylation, histone modification and non-coding RNA are major epigenetic mechanisms that regulate gene transcription in carcinogenesis [54]. It is currently accepted that these epigenetic modifications are linked to one another in the modulation of the epigenome landscape [55, 56]. For example, these epigenetic modifications may work in combination in carcinogenesis [57]. It was found that DNA hypermethylation in Apc mutant adenomas preferentially target the polycomb repressive complex 1/2 (PRC 1/2) target genes, suggesting an interplay of DNA methylation and histone modification in Apc^min/+^ mice [27]. On the other hand, different epigenetic mechanisms may cross-regulate each other in the regulation of cellular activity. For instance, the expression of certain microRNAs is potentially controlled by DNA methylation or histone modification. However, some microRNAs can target epigenetic-modifying enzymes, such as DNMTs (DNA methyltransferases) and EZH2 (enhancer of zeste homolog 2) [58]. Furthermore, Tahara, et al. found that 74 chromatin regulatory genes are mutated more frequently in CpG island methylator phenotype - high CRC in the TCGA dataset [59]. Changes in the methylation patterns of several genes encoding microRNAs, histone modification enzymes, and proteins that function in chromatin remodeling were identified using MeDIP-seq. For example, we discovered decreased methylation of microRNA-155 (log_2_ fold change = −2.7, Table 5), which mapped to the EMT pathway; microRNA-155 expression promotes the migration and invasion of several CRC cell lines [60]. Moreover, HDAC1 (histone deacetylase 1) was mapped to the Wnt/β-catenin pathway with a 2.9-fold (log_2_) increase in methylation in Apc mutant polyps (Table 6). In addition, we observed an increased methylation in the gene coding for chromodomain-helicase-DNA-binding protein 1 (CHD1) in Apc mutant polyps (data not shown). CHD1 protein is known to be involved in transcription-related chromatin remodeling [61]. Taken together, our data indicated that epigenetic alterations may be complex and may occur at multiple levels during tumorigenesis in Apc^min/+^ mice.

## Conclusions

In conclusion, polyps from Apc^min/+^ mice exhibited extensive, aberrant DNA methylation. The methylation changes in the genes detected using the MeDIP-seq assay were mainly attributed to functions and networks in cancer, the cell cycle, and gastrointestinal diseases. These differentially methylated genes were situated in several canonical pathways that are important in colon cancer, such as the EMT and Wnt/β-catenin signaling pathways.

## Materials and methods

### Mouse strains

C57BL/6 J male mice that are heterozygous for the Apc allele (Apc^min/+^) and their wild type littermates (Apc^+/+^) were originally obtained from Jackson Laboratories (Bar Harbor, ME, USA). The animals were housed in the Animal Care Facility at Rutgers University with a 12 h-light/12 h-dark cycle and were provided ad libitum access to food and water. The Apc^min/+^ and control mice were sacrificed by CO_2_ inhalation at 20 weeks of age. Polyp and intestine samples were collected as previously described [62]. Briefly, after sacrificing the mice, the gastrointestinal tract was removed, opened longitudinally, and rinsed thoroughly with saline. Intestinal adenomatous polyps were excised from the intestines carefully. The normal intestine tissue and polyps were snap frozen and stored at −80 °C for future use.

### DNA extraction

Genomic DNA was isolated from adenomatous polyps from three Apc^min/+^ mice and from normal intestinal tissue from three Apc^+/+^ littermates using a DNeasy Kit (Qiagen, Valencia, CA, USA). Prior to fragmentation by Covaris (Covaris, Inc., Woburn, MA, USA), the quality of the extracted genomic DNA was confirmed by agarose gel electrophoresis and OD ratio. After fragmentation, the genomic DNA was further assessed for size distribution using an Agilent Bioanalyzer 2100 (Agilent Technologies, Santa Clara, CA, USA). The fragmented genomic DNA concentrations were measured with a Nanodrop spectrophotometer.

### MeDIP-seq

MeDIP was performed using a MagMedIP kit (Diagenode, Denville, NJ, USA) as previously described [63]. Briefly, immunoprecipitations were performed using a monoclonal antibody against 5-methylcytidine (Diagenode, Denville, NJ, USA) to separate the methylated DNA fragments from the unmethylated fragments. The captured DNA was used to create the Illumina libraries using NEBNext reagents (catalog# E6040; New England Biolabs, Ipswich, MA, USA). After the quality of the libraries was evaluated, the samples were sequenced using an Illumina HiSeq 2000 machine. The results were analyzed for data quality and exon coverage using the platform provided by DNAnexus (DNAnexus, Inc., Mountain View, CA, USA). Subsequently, the samples were subjected to Illumina next-generation sequencing (Otogenetics Corporation, Norcross, GA, USA). After downloading the BAM files for analysis, MeDIP alignments were compared with control samples using Cuffdiff 2.0.2 as previously described [64, 63]. To judge the quantitative enrichment in MeDIP samples versus control samples in Cuffdiff, the overlapping regions of sequence alignment common to the MeDIP and control samples were used. Significant peaks at a 5 % false discovery rate (FDR) with a minimum of a 4-fold difference in R (Cummerbund package) were selected. The peaks were matched with adjacent annotated genes using ChIPpeakAnno as previously described [65].

### Ingenuity Pathway Analysis (IPA)

To investigate the significance of the altered methylation observed by MeDIP-seq, we analyzed genes that exhibited greater than a 2-fold change (log_2_) in methylation (Apc^min/+^ polyps vs. control) using IPA (IPA 4.0, Ingenuity Systems, www.ingenuity.com). IPA utilized gene symbols that were identified as neighboring enriched methylation peaks by ChIPpeakAnno for all of the analyses. IPA mapped the input genes to its knowledge bases and identified the most relevant biological functions, networks, and canonical pathways related to the altered methylation profiles in the Apc mutant polyps.
